# Probiotics and Their Role in the Management of Type 2 Diabetes Mellitus (Short-Term Versus Long-Term Effect): A Systematic Review and Meta-Analysis

**DOI:** 10.7759/cureus.46741

**Published:** 2023-10-09

**Authors:** Ismat E Ayesha, Neetha R Monson, Nimra Klair, Utkarsh Patel, Ayushi Saxena, Dhara Patel, Sathish Venugopal

**Affiliations:** 1 Internal Medicine, California Institute of Behavioral Neurosciences & Psychology, Fairfield, USA

**Keywords:** homeostasis model assessment for insulin resistance (homa-ir), glycated hemoglobin (hba1c), fasting blood glucose (fbg), diabetes mellitus type 2, glycated hemoglobin, randomized controlled trials, probiotics

## Abstract

Diabetes is a major economic burden and an illness with a rising incidence worldwide. Type 2 diabetes mellitus (T2DM), the most prevalent kind of diabetes, is characterized by insulin resistance and insufficient insulin production. Recent research has implicated gut microbiota dysbiosis as a contributing factor to T2DM pathogenesis. The present study employed a methodology based on randomized controlled trials (RCTs) to assess the therapeutic efficacy of probiotics in the treatment of T2DM. A thorough search was done in PubMed and Medline for articles written in English and published between 2017 and 2023. Studies were chosen based on predetermined inclusion criteria, and the search technique adhered to Preferred Reporting Items for Systematic Reviews and Meta-Analyses (PRISMA) principles. This study also employed a robust assessment instrument, widely recognized in the medical and health sciences, to evaluate the potential presence of bias within the selected research studies. Out of 96 identified articles, 22 RCTs met the eligibility criteria. Both short-term (8 weeks or less) and long-term (12 weeks or more) probiotic administrations were made. The results of the meta-analysis demonstrated a significant improvement in the homeostatic model assessment of insulin resistance (HOMA-IR) following the probiotic intervention (P=0.02) and considerably decreased glycated hemoglobin HbA1c levels (P=0.004) and fasting blood glucose (FBG) levels (P<0.0001) in T2DM patients compared to placebo. This research offers proof that probiotics are clinically effective in the treatment of T2DM. Probiotic supplementation demonstrated favorable effects on glycemic control markers. However, the findings from RCTs were heterogeneous, and some studies showed inconsistent results. To clarify the processes underlying the probiotics' therapeutic benefits and to determine the best probiotic strains, doses, and therapy durations, more research is required. Nevertheless, probiotics offer a promising therapeutic approach for T2DM management and warrant consideration as a potential adjunct therapy in clinical practice.

## Introduction and background

Increased blood glucose levels are a hallmark of the chronic metabolic condition known as diabetes mellitus. The manifestation of this condition occurs when the pancreatic gland fails to secrete an adequate amount of insulin or when the body exhibits an impaired ability to utilize the insulin that is produced. Diabetes mellitus, a metabolic disorder characterized by chronic hyperglycemia, has emerged as a prominent health concern in the contemporary era. Its prevalence has witnessed a rapid escalation on a global scale, rendering it one of the foremost health challenges of the 21st century. By 2045, 700 million individuals worldwide are expected to have diabetes mellitus, up from 463 million in 2019. The global health expenditure on diabetes management was nearly 760 billion USD in 2019, which will continue to increase with the escalating diabetes prevalence. Thus, diabetes mellitus is a quickly growing public health problem and economic burden [1]. Type 2 diabetes mellitus (T2DM) is the prevailing manifestation of diabetes, constituting approximately 90% of the global incidence of this disease. It is characterized by early insulin resistance-related hyperglycemia. As a result, there is a hypersecretion of insulin to overcome the inadequate insulin response. In the long run, this leads to inadequate insulin production, and the pancreatic cells fail to comply with the increased demand [1]. The resulting continuous hyperglycemia in the body affects the human vasculature directly as well as indirectly, leading to microvascular as well as macrovascular complications. The main factor of morbidity and death in T2DM is these comorbidities [2].

T2DM is typically a consequence of a mixture of different hereditary, metabolic, and ecological elements. The well-known risk factors contributing to T2DM include genetic susceptibility via solid family history, obesity, age, physical inactivity, and unhealthy dietary habits (high-calorie food, lack of a balanced diet, etc.) [3]. The gut microbiota has emerged as a potential, influential element in the underlying pathophysiology of obesity and T2DM over the past decade. The human gastrointestinal system harbors a vast population of bacteria, numbering in the millions, with a particular abundance observed in the distal gut region [4]. These microbes, which together weigh close to 1500 g, may be considered a microbial organ that carries out crucial tasks that the human body is unable to do on its own, e.g., digestion, extracting energy from food products, production of vitamins, xenobiotic metabolism, production of metabolites and antioxidative role performed through the production of reactive oxygen species scavengers, metal ion chelators, enzyme inhibitors, and reducers [4-6].

Species from all three domains of life, bacteria, eukaryotes, and archaea, comprise the gut microbiota [4]. The 16S ribosomal ribonucleic acid (rRNA) gene amplicon sequencing and shotgun metagenomics sequencing, two deoxyribonucleic acid (DNA)-based, culture-independent sequencing techniques, have improved our understanding of gut microbiota [7]. According to the metagenomic studies, 90% of the bacteria inhabiting the human intestine are members of Bacteroidetes and Firmicutes phyla. Furthermore, bacteria belonging to phyla like Proteobacteria, Actinobacteria, and Verrucomicrobia have a low abundance [8]. However, a condition known as dysbiosis can result from external causes such as food, the use of antibiotics, stress, gut inflammation, toxins, and other conditions [9]. According to studies conducted on both humans and animals, significant dysbiosis may be linked to obesity and T2DM. However, data from human investigations showed that T2DM patients' gut microbiota composition differs significantly from that of non-diabetic control people. Although the studies did not all agree on the precise microbiota makeup, they were all distinguished by a reduction in bacteria that produced butyrate [10-12].

Through a variety of mechanisms, including increased energy extraction from food, distorted fatty acid metabolism, particularly short-chain fatty acids (SCFAs), altered adipose tissue composition and sensitivity to insulin, metabolic endotoxemia, increased systemic inflammation, and intestinal permeability, human research and animal models have linked this change in gut microbiota to the pathogenesis of obesity, insulin resistance, and subsequently T2DM [10-13]. It is thus hypothesized that the gut microbiota significantly contributes to the onset and progression of T2DM [14].

It is possible to alter the gut microbiota for the better by employing probiotics, prebiotics, or fecal microbiota transplantation (FMT). The utilization of probiotics as functional foods and dietary supplements is gaining popularity [14]. They are characterized as being alive microorganisms that, if consumed properly, can enhance the host's health. The preclinical data from animal and human studies have shown an evident beneficial effect of probiotics in improving glycemia and other associated metabolic factors in T2DM patients [15-18]. Nevertheless, the outcomes of various randomized controlled trials (RCTs) have presented inconclusive results regarding the efficacy of probiotics in the management of T2DM [19-22]. Previous studies have conducted a multitude of systematic reviews and meta-analyses to elucidate the immediate impacts of probiotics on individuals diagnosed with T2DM; there is at least one systematic review on the long-term effect of this proposed treatment in humans [22-25]. This article aims to review the clinical evidence present in the form of RCTs on the clinical efficacy of probiotics’ use in T2DM patients while comparing its short-term effect (taken as 8 weeks or less) versus the long-term (taken as 12 weeks or more). Fasting blood glucose (FBG), glycated hemoglobin (HbA1c), and homeostatic model assessment of insulin resistance (HOMA-IR) are the three parameters used for the comparison in these studies.

## Review

Method

Study Design

A search for relevant English-language full-text articles published from 2017 to 2023 was effectuated in online literature databases including Medline and PubMed. The study question was created using the Problem/Population, Intervention, Comparison, Outcome (PICO) format, as advised by Cochrane and Preferred Reporting Items for Systematic Reviews and Meta-Analyses (PRISMA) guidelines [26]. A search approach that incorporates all pertinent articles was developed using terms from the Medical Subject Headings (MeSH) and keywords from the pertinent literature. Search parameters were [Probiotics OR symbiotic] AND [Type 2 Diabetes Mellitus OR Diabetes (T2DM) OR T2DM] AND [short-term effect OR long-term effect]. A cross-referencing procedure was conducted in the reference lists of the included articles to identify additional relevant research. The PRISMA standards were followed for conducting this study [27]. The scientists separately decided whether or not to include the titles and abstracts. A critical appraisal methodology was employed in the field of medical and health sciences to evaluate the presence of bias in the selected papers.

Study Inclusion and Exclusion Criteria

The authors independently examined the titles and abstracts to identify articles that would be possibly appropriate for full-text examination. The same process was followed throughout the whole text review. Reference lists from included papers were also checked to find other, perhaps suitable studies. Studies were only deemed qualified if they complied with the inclusion criteria (Table 1).

**Table 1:** Inclusion and exclusion standards for the present research T2DM: type 2 diabetes mellitus; HOMA-IR: homeostatic model assessment of insulin resistance; FBG: fasting blood glucose; HbA1c: hemoglobin A1c

Components	Criteria for inclusion	Criteria for exclusion
Population	T2DM patients	Patients having disease other than T2DM
Intervention	Studies on the utilization of probiotics for the treatment/management of T2DM patients, with patients not on insulin	Anything relating to T2DM patient management not covered by the subjects listed
	All sorts of study designs, including mixed methods, quantitative, and qualitative, had been subject to peer review.	Anything except works that had undergone peer review, including reviews, blogs, book chapters, website material, and more.
	Original articles	Reviews
	English-language studies	Publications in other languages
Comparator/control	With a placebo control group	Studies with no control group
Outcome	HOMA-IR, FBG, and HbA1c	Studies reporting no outcome

Data Extraction and Analysis

The authors independently gathered information on the author, publication year, country, patients, type of probiotics, duration, and outcomes. The two reviewers resolved discrepancies in data gathering using the original publications as a reference; if no consensus could be reached, they were referred to a third reviewer.

Results

**Figure 1:**
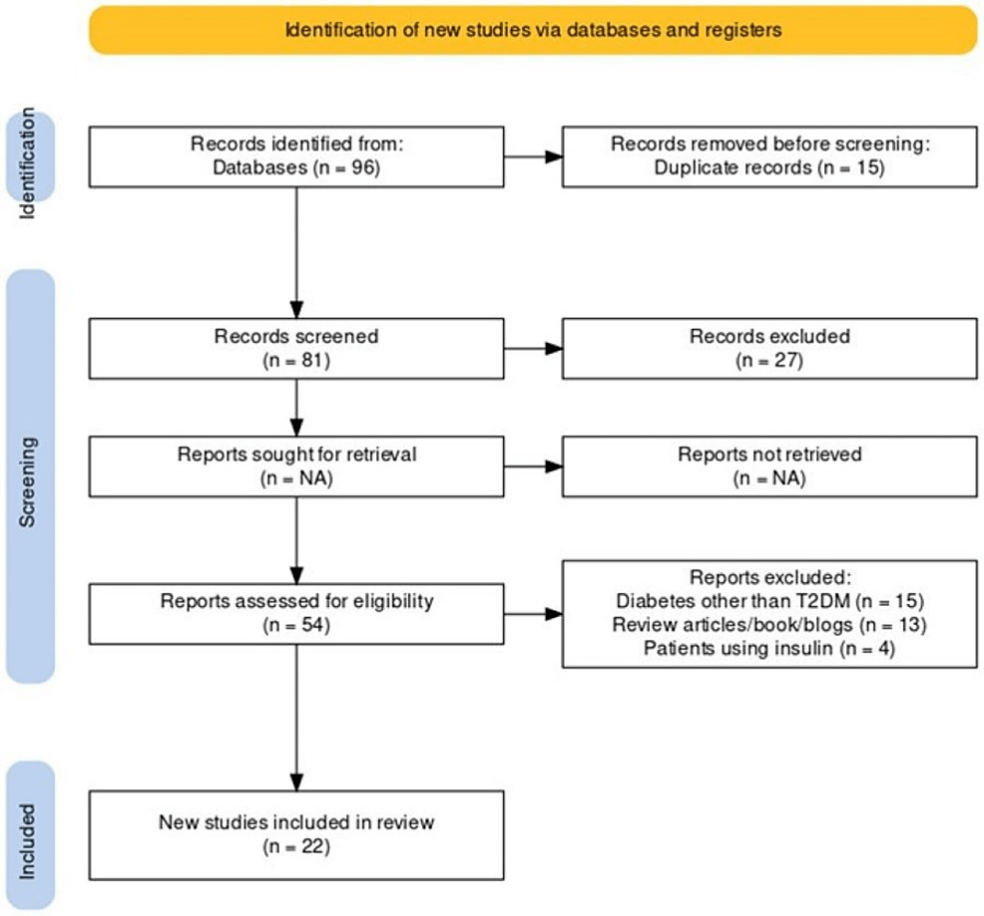
PRISMA flowchart for choosing appropriate literature for meta-analysis PRISMA: Preferred Reporting Items for Systematic Reviews and Meta-Analyses; n: number of articles; T2DM: type 2 diabetes mellitus; NA: not applicable

A total of 96 articles were revealed after the original search, and 15 duplicate records were removed. Following an examination of the publications' titles and abstracts, 27 were eliminated from the study. The remaining 54 papers underwent thorough examination and further screening based on research. A total of 22 studies with investigations on probiotics and their role in the management of T2DM were found to be eligible (Figure 1).

**Figure 2:**
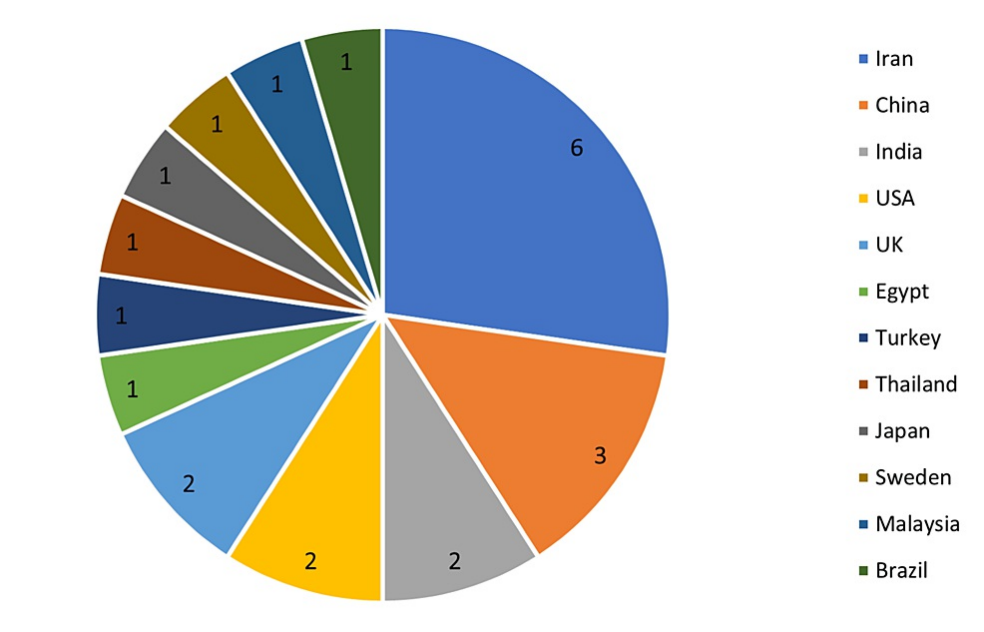
Distribution of chosen studies by country

Iran had the maximum number of chosen studies (n=6), followed by China (n=3), India (n=2), United States (n=2), and the United Kingdom (n=2), while one study each from Egypt, Turkey, Thailand, Japan, Sweden, Malaysia, and Brazil was selected (Figure 2).

Five of the selected studies compared short-term effects (taken as 8 weeks or less) of probiotics while 17 studies compared long-term effects (taken as 12 weeks or more) of probiotics. Kumar et al. used a probiotic capsule and reported that it reduced the levels of HbA1c, postprandial blood glucose, and FBG, whereas Şahin et al. used *Bifidobacterium animalis* subsp. *lactis* (BB-12) for 12 weeks and observed better glycemic control and better HbA1c reduction [28,29]. Similarly, Ismail et al. also utilized *B. animalis* dn-173 010 for 16 weeks and saw favorable effects on lipid profile, inflammatory markers, and glycemic management [30]. Another selected study by Toejing et al. also used single *Lactobacillus paracasei* HII01 strain for 12 weeks and suggested its possible use as an adjuvant therapy for T2DM [31]. Sanborn et al. used *Lactobacillus rhamnosus* GG for 12 weeks and reported alterations in blood sugar regulation [32]. Eight of the evaluated studies utilized a single probiotic strain to treat T2DM, whereas the other 13 trials used several probiotic strains. The characteristics of selected studies are presented in Table 2.

**Table 2:** Characteristics and findings of selected studies NM: not mentioned; T2DM: type 2 diabetes mellitus; FBG: fasting blood glucose; HOMA-IR: homeostatic model assessment of insulin resistance; HbA1c: hemoglobin A1c; SCFA: short-chain fatty acid

Author (year)	Patients	Probiotics	Duration (weeks)	Outcomes
Kumar et al. (2022) [28]	150	NM	12	In the context of treating T2DM, the addition of probiotics to metformin as a supplementary therapeutic approach was found to result in reductions in FBG, postprandial blood glucose, and levels of HbA1c when compared to the use of metformin alone. The efficacy of probiotics in combination treatment has not been substantiated by significant findings. Nonetheless, the probiotics trial group exhibited a reduced incidence of gastrointestinal side effects associated with metformin therapy.
Şahin et al. (2022) [29]	156	*Bifidobacterium animalis* subsp. *lactis* (BB-12)	12	Patients receiving probiotic supplements showed improved glycemic control and HbA1c decrease, as well as improved treatment compliance and potential effects on the intestinal-pancreatic axis.
Ismail et al. (2021) [30]	150	*Bifidobacterium* *animalis* dn-173 010	16	After 16 weeks, probiotic use in T2DM patients improved glycemic control, lipid profile, and inflammatory markers.
Toejing et al. (2021) [31]	50	*Lactobacillus paracasei* HII01	12	*L. paracasei *HII01 reduced inflammatory indicators and hyperglycemia by properly regulating the stomach microbiota and so treating endotoxemia and damaged stomach, proposing a possible role as an adjuvant therapy for T2DM.
Sanborn et al. (2020) [32]	200	*Lactobacillus rhamnosus* GG	12	HbA1c was steady in individuals taking *L. rhamnosus* GG, whereas it rose in those receiving placebo at the follow-up. *L. rhamnosus* GG may offer a defense against alterations in blood sugar regulation.
Chen et al. (2023) [33]	58	*Bifidobacterium animalis* subsp. *lactis* M8, *B. animalis* subsp. *lactis* V9, *Lactobacillus casei* Zhang, *L. plantarum* P-8, and *L. rhamnosus* Probio-M9	12	The findings of this study demonstrated that the co-administration of probiotics and metformin in individuals with T2DM resulted in an augmented hypoglycemic response. The observed impact is likely to have been facilitated through the modulation of the gastrointestinal microbiota, subsequently influencing the metabolism of bile acids and SCFAs. This study provides evidence supporting the benefits of combining metformin and probiotics as a treatment approach for individuals diagnosed with T2DM.
Hasanpour et al. (2023) [34]	100	*Bifidobacterium longum*, *B. breve*, *Lactobacillus* *bulgaricus*, *L. rhamnosus*, *L. casei*, *L. acidophilus*, and *Streptococcus thermophilu*	6	Consuming soymilk and probiotics may reduce several cardiovascular risk factors in T2DM patients. FBG and HOMA-IR were not considerably impacted, though.
Velayati et al. (2021) [35]	50	*Lactobacillus* *rhamnosus*, *Bacillus coagulans*, fructooligosaccharide and *L. acidophilus*	12	This study showed that symbiotic *B. coagulans* supplementation might reduce metabolic variables and inflammation in T2MD individuals.
Kanazawa et al. (2021) [36]	88	*Bifidobacterium breve* strain Yakult, *Lacticaseibacillus paracasei *strain Shirota, and galactooligosaccharides	24	Regarding incendiary markers, there was no method to distinguish between the treatment groups. The stomach environment was undoubtedly slightly altered by synbiotic treatment in obese individuals with T2DM.
Jiang et al. (2021) [37]	101	*Bifidobacterium* *bifidum*, *Lactobacillus* *acidophilus*, *Streptococcus thermophilus*	12	This clinical investigation found that administering probiotics in individuals with diabetic nephropathy improved their glycemic control, amplifying their therapeutic potential in clinical settings.
Ming et al. (2021) [38]	300	*Bifidobacterium* and berberine	16	According to this study, *Bifidobacterium* may improve the hypoglycemic effects of berberine.
Perraudeau et al. (2020) [39]	76	*Clostridium butyricum*, *Akkermansia muciniphila*, *C. beijerinckii*, *Anaerobutyricum hallii* and *Bifidobacterium infantis*	12	In participants with T2DM who were predominantly receiving metformin monotherapy, a unique five-strain probiotic formulation decreased total glucose in comparison to placebo. No changes in the body weight, HOMA-IR, or fasting glucose levels were seen, indicating that the majority of the impact was a decrease in FBG levels during the postprandial period.
Khalili et al. (2019) [40]	40	*Lactobacillus* *casei*	8	*L. casei* supplementation altered serum sirtuin 1 (SIRT1) and fetuin-A levels in people with T2DM in a manner that enhanced glycemic response. A novel recognised method of probiotic action in the treatment of diabetes was introduced by altering their amounts.
Razmpoosh et al. (2019) [41]	60	*Lactobacillus*, *Bifidobacterium*, and *Streptococcus*	6	This study found that using multi-strain probiotic supplements significantly reduced fasting plasma glucose levels when compared among groups, although more research is required to validate the findings.
Madempudi et al. (2019) [42]	79	*Lactobacillus* *casei* UBLC42, *L. acidophilus *UBLA34, *L. plantarum *UBLP40, *Bacillus* *coagulans* Unique IS2, *Bifidobacterium breve* UBBr01 and fructo-oligosaccharides, *L. salivarius* UBLS22	12	The reduction in HbA1c values showed that UB0316 (probiotic) considerably improved glycemic management. Additionally, the probiotic-treated patients' weight significantly decreased as compared to the control group.
Sabico et al. (2019) [43]	150	*Bifidobacterium* *bifidum* W23, *Lactobacillus* *brevis* W63, *L. acidophilus* W37, *L. salivarius* W24, *L. casei* W56, *Lactococcus lactis* W58, *Lactococcus* *lactis* W19, and *B. lactis* W52	24	In T2DM patients, six months of multi-strain probiotic treatment as a monotherapy dramatically decreased HOMA-IR. Consequently, multi-strain probiotics are a successful adjunctive treatment for diabetes.
Mafi et al. (2018) [44]	60	*Lactobacillus *with *Bifidobacterium*	12	Supplementing with probiotics improved indicators of cardiometabolic risk and glycemic control.
Mobini et al. (2017) [45]	46	*Lactobacillus* *reuteri* DSM 17938	12	The administration of *L. reuteri* DSM 17938 for a duration of 12 weeks did not result in any significant changes in HbA1c levels among individuals with T2DM who were undergoing insulin treatment. Nevertheless, the impact of *L. reuteri* on the insulin response was observed to be significant only in a limited subset of individuals, leading us to hypothesize that this discrepancy could potentially be attributed to the diverse compositions of gut microbiota.
Sabico et al. (2017) [46]	78	*Bifidobacterium* *lactis* W52, *B. bifidum *W23, *Lactobacillus* *brevis* W63, *L. acidophilus* W37, *L. salivarius* W24, *L. casei *W56, *Lactococcus lactis* W58, and *Lactococcus lactis* W19	12	A multi-strain probiotic supplement was taken by T2DM patients who had not taken any medication for 12 or 13 weeks. This greatly improved HOMA-IR and very slightly decreased abdominal obesity.
Firouzi et al. (2017) [47]	136	*Lactobacillus* and *Bifidobacterium*	12	In persons with type 2 diabetes, probiotics only slightly decreased HbA1c and fasting insulin levels.
Feizollahzadeh et al. (2017) [48]	40	*Lactobacillus plantarum* A7	8	Utilising probiotic soy milk did not decrease inflammation or serum adiponectin; however, it could alter a patient's lipid profile if they had T2DM.
Tonucci et al. (2017) [49]	50	*Bifidobacterium animalis* subsp. *lactis* BB-12 and *Lactobacillus acidophilus* La-5	6	Probiotics reduced T2DM patients' glucose levels across the board, but consumption of mature milk appears to be linked to additional metabolic changes, including a decline in pro-inflammatory cytokines and an increase in acidic corrosive enzymes.

**Figure 3:**
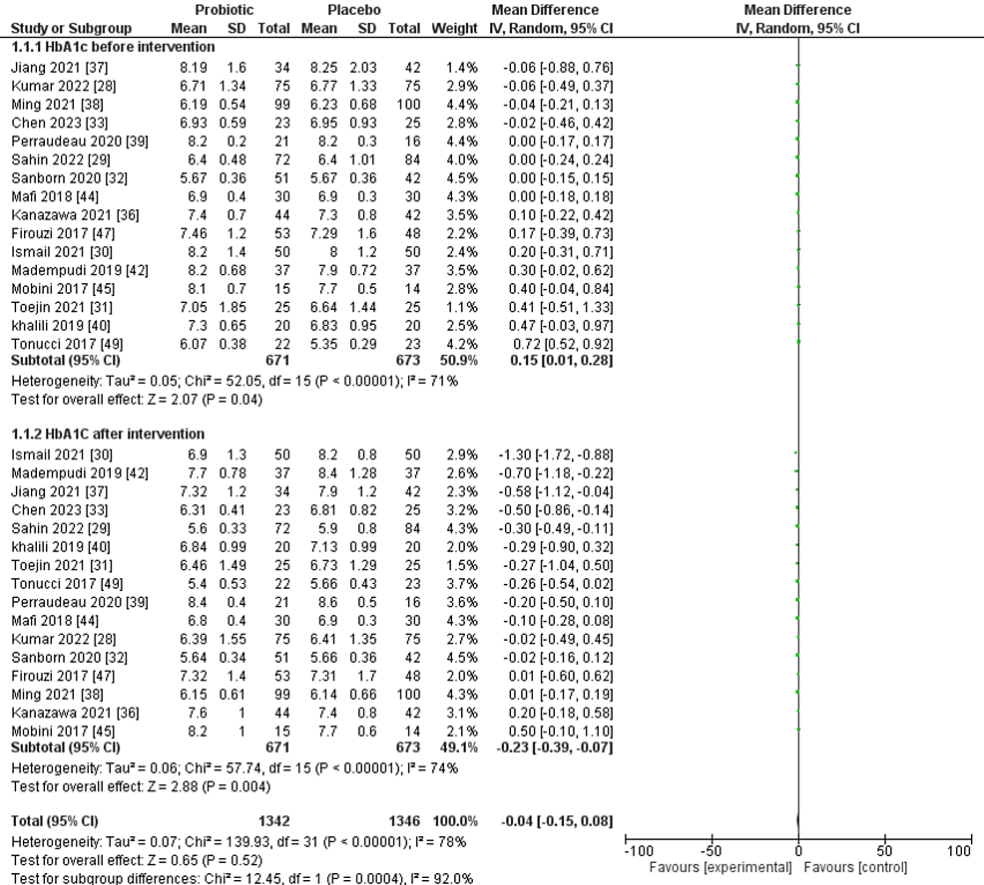
A forest plot comparing probiotic and placebo groups before and after the intervention to assess HbA1c SD: standard deviation; IV: interval; CI: confidence interval; HbA1c: hemoglobin A1c, df: difference Source: [28-33,36-40,42,44,45,47,49]

In 16 of the selected trials, the results of glycated hemoglobin (HbA1c) were observed both prior to and following the implementation of the intervention. Across all selected trials, the probiotic group consisted of 671 patients, while the placebo group comprised 673 individuals. The forest plot for HbA1c before intervention showed 71% I^2^ value for heterogeneity among studies. Before the intervention, there was just a little difference (P=0.04) between the probiotic and placebo groups; however, the forest plot showed a highly significant difference (P=0.004) with more beneficial effects observed in patients in the probiotic group. Figure 3 displays the findings of a meta-analysis comparing the effects of probiotic and placebo interventions on HbA1c levels.

**Figure 4:**
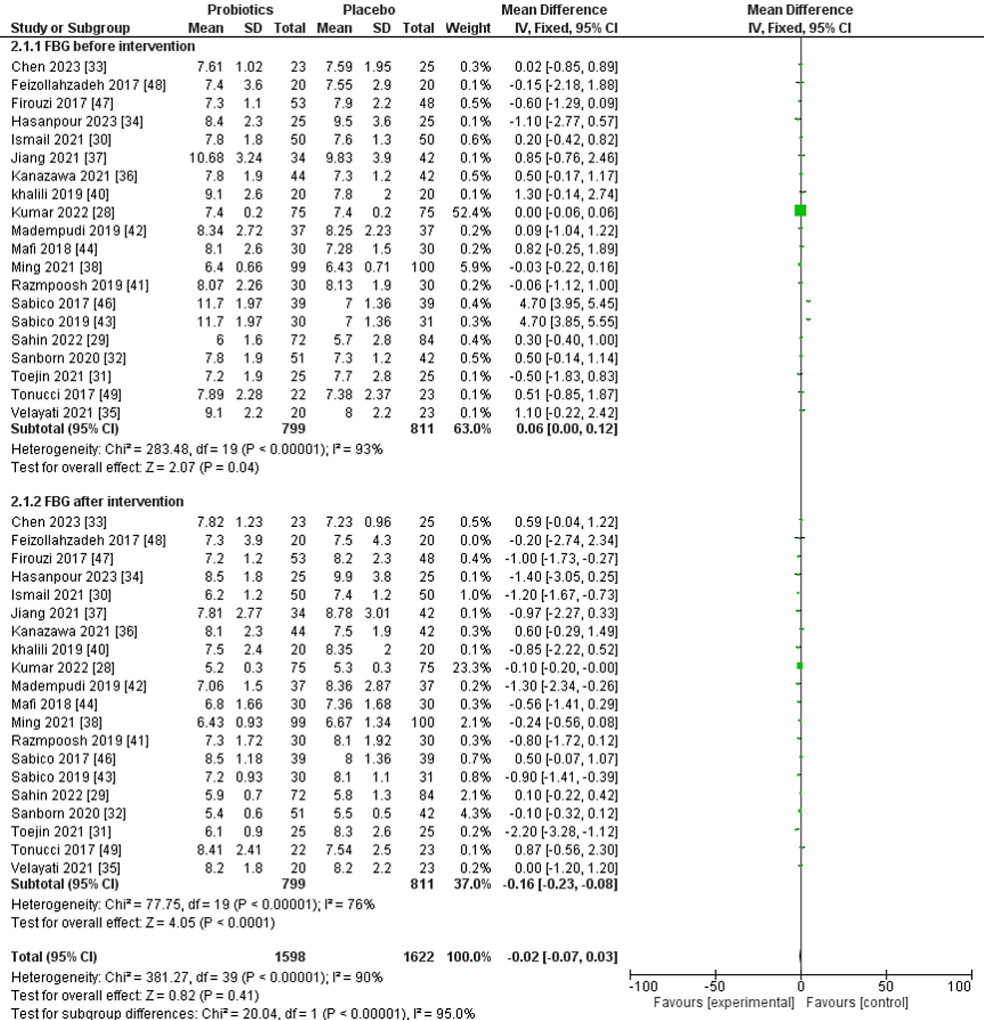
A forest plot comparing probiotic and placebo groups before and after the intervention to assess FBG SD: standard deviation; IV: interval; CI: confidence interval; FBG: fasting blood glucose; df: difference Source: [28-38,40-44,46-49]

In 20 of the chosen trials, the results of FBG were observed both before and after the intervention. Across all selected trials, the probiotic group consisted of 799 patients, while the placebo group comprised 811 patients. The forest plot for FBG before the intervention showed 93% I^2^ value for heterogeneity among studies. Overall, there was a slight difference (P=0.04) between placebo and probiotic groups prior to the intervention; however, the forest plot showed a highly significant difference (P<0.0001) with more beneficial effects observed in patients in the probiotic group. Figure 4 shows outcomes of a meta-analysis comparing the effects of probiotic and placebo interventions on FBG before and after the intervention.

**Figure 5:**
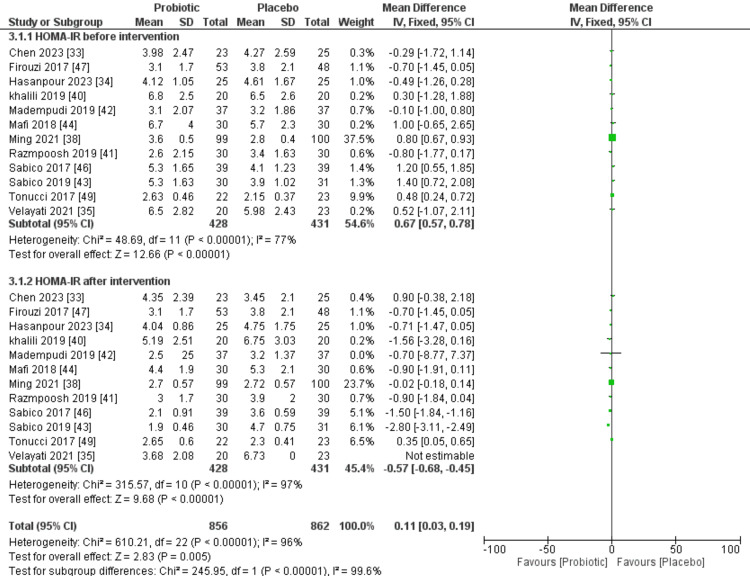
A forest plot comparing probiotic and placebo groups before and after the intervention to assess HOMA-IR SD: standard deviation; IV: interval; CI: confidence interval; HOMA-IR: homeostatic model assessment of insulin resistance; df: difference Source: [33-35,38,40-44,46,47,49]

Twelve of the selected studies presented HOMA-IR outcomes before and after the intervention. In each of the selected trials, the probiotic group consisted of 428 patients, while the placebo group comprised 431 patients. The forest plot for HOMA-IR before the intervention showed 77% I^2^ value for heterogeneity among studies. Overall, there was a non-significant difference (P=0.07) between placebo and probiotic groups prior to the intervention; however, the forest plot showed a highly significant difference (P=0.02) with more beneficial effects observed in patients in the probiotic group. Figure 5 shows the outcomes of the meta-analysis comparing the effects of probiotic and placebo interventions on HOMA-IR.

Discussion

In the current investigation, we systematically reviewed the literature and checked the effects of probiotics in T2DM patients. This study comprised 22 RCTs totaling 2218 patients. The findings suggested that probiotics may lower baseline levels of HbA1c, FBG, and HOMA-IR. Gut microbiota significantly contributes to T2DM [50]. For treating T2DM and associated consequences, probiotic and synbiotic supplementation has drawn a lot of interest. For instance, according to a research by Łagowska et al., the administration of synbiotics and probiotics has been observed to have a beneficial effect on reducing blood glucose levels, HOMA-IR, and insulin levels in pregnant women diagnosed with gestational diabetes mellitus [51]. Synbiotics and probiotics both lower HOMA-IR and serum insulin levels, yet just probiotics have the ability to bring down glucose. Another study recommends that synbiotic and probiotic supplementation might be useful in bringing down FBG levels in people with high baseline FBG [52]. To evaluate the impacts of synbiotics, Tabrizi et al. carried out an investigation of seven RCTs and found that synbiotics improved the patients' condition with T2DM [53]. Rittiphairoj et al. discovered that probiotics may reduce blood sugar in T2DM patients without changing HbA1c levels in another meta-analysis of 28 trials [22]. In this study, only long-term focus areas revealed beneficial effects of probiotic supplementation. According to Du et al., synbiotic and probiotic supplementation enhanced glycemic control variables in people with prediabetes [54].

In T2DM patients, the HbA1c level can accurately indicate how well the patients' blood glucose is being controlled. Patients' HbA1c levels are determined by both fasting and postprandial blood glucose, with the latter considerably increasing HbA1c [55]. The impact of probiotics on HbA1c percentage was quite significant in our meta-analysis. It could be brought on by an unidentified postprandial blood glucose level that was left out of the evaluation due to insufficient clinical information. In one of the researches, it was found that those with prediabetes and T2DM who got probiotics and metformin together rather than metformin alone saw significant reductions in HbA1c levels and gastrointestinal intolerance [29]. Zhang et al. reported similar results in a meta-analysis [56]. Intestinal microorganisms produce short-chain fatty acids, byproducts that manage immune reaction, gluconeogenesis, modify gastrointestinal hormonal secretion, decrease gut permeability, maintain intestinal anaerobic environment, and mediate glucose homeostasis by furnishing colonocytes with energy [50,57]. Probiotics may exercise their glycemic-moderating effects via reducing inflammation and oxidative stress in hyperglycemic individuals. From a sub-atomic perspective, oxidative stress and inflammation have been linked to increased insulin resistance, decreased glucose resilience, and mitochondrial cell dysfunction [58,59]. Last but not the least, probiotics and synbiotics have been found to have antioxidant properties [60]. Additionally, antioxidants are believed to be able to control insulin resistance [61].

In addition to the fact that probiotics reduced blood sugar, our selected studies showed that these also have a role in reducing inflammation. Obesity is a risk factor for developing insulin resistance in T2DM patients, but intriguingly, without changing the body mass index (BMI), probiotics were found to decrease insulin resistance [47,49]. Our meta-analysis revealed a minimal disparity between the probiotic and placebo cohorts prior to the implementation of the FBG intervention. However, upon examination of the forest plot, a substantial discrepancy emerged, indicating a significantly greater magnitude of positive outcomes among patients assigned to the probiotic group. Prior to the implementation of the intervention, there was no discernible distinction between the groups receiving probiotics and those receiving a placebo in terms of the HOMA-IR. However, upon analysis using a forest plot, a remarkably significant disparity was observed, with a greater number of favorable outcomes observed among patients in the probiotic group. In one of the studies, Ismail et al. used *B. animalis* dn-173 010 and reported beneficial effects on inflammatory markers, lipid profile and glycemic control, after 16 weeks [30]. Another study by Toejing et al. also used single *L. paracasei* HII01 strain for 12 weeks and reported its potent role as an adjuvant treatment in T2DM [31]. Sanborn et al. used *L. rhamnosus* GG for 12 weeks and reported alterations in blood sugar regulation [32]. In a research by Andreasen et al., individuals received an intravenous injection of *Escherichia coli* lipopolysaccharide (LPS) two days after the probiotic intervention [62]. We hypothesize that the improvement in the gut microbiota and the decrease in the blood's exposure to LPS contributed to how effectively probiotics cured insulin resistance in vivo. Additionally, the reduction in insulin resistance may contribute to the decline in FBG and HbA1c%. These factors lead us to hypothesize that T2DM was not caused by patients' abnormal gut flora.

Strengths and Limitations of the Study

This study followed a systematic approach, including a well-defined search strategy, clear inclusion and exclusion criteria, and adherence to PRISMA standards. This enhances the reliability and reproducibility of the findings. This study also considered a diverse set of studies published over a span of years, contributing to a comprehensive overview of the current literature on probiotics and T2DM. Furthermore, by differentiating between short-term and long-term interventions, the study provides insights into the potential timing-dependent impacts of probiotics on T2DM indicators. However, this study also has some limitations that include heterogeneity among studies that affected the ability to draw consistent conclusions. There might be a bias towards publishing studies with positive outcomes, potentially leading to an overrepresentation of studies showing significant effects of probiotics on T2DM management. The inclusion of relatively short follow-up periods, especially in the short-term interventions lasting six weeks or less, poses a limitation in evaluating the long-term effects of probiotics on T2DM indicators. T2DM is a chronic condition that develops over years, so short-term assessments may not capture the full spectrum of how probiotics affect the disease, emphasizing the necessity for longer term studies to comprehensively assess their sustained impact on T2DM management. Variability in study participant numbers in research on probiotics and T2DM can impact statistical power, with smaller samples potentially missing important effects, and generalizability, as smaller samples may not represent the broader population accurately. Larger, well-powered studies are essential for robust and widely applicable findings in this field. The wide-ranging use of various probiotic strains across studies presents a challenge in pinpointing the most effective strains for T2DM management. This diversity complicates efforts to establish clear recommendations regarding which specific strains are the most beneficial in the context of T2DM, underscoring the need for further research and standardization in probiotic interventions for T2DM.

## Conclusions

This systematic review indicates that probiotics have the potential to lower baseline levels of HbA1c, FBG, and HOMA-IR in T2DM patients. Furthermore, the study highlights the significant contribution of gut microbiota to the development of T2DM, with dysbiosis potentially linked to obesity and insulin resistance. Selected studies suggest that specific strains of probiotics, such as *L. rhamnosus* GG and *B. animalis*, could serve as adjuvant therapies for T2DM management. In conclusion, our findings suggest that incorporating probiotics into T2DM management strategies could offer potential benefits in terms of glycemic control, insulin sensitivity, and inflammation reduction. More research studies, particularly randomized controlled trials, are required to establish conclusive evidence and determine optimal probiotic strains and dosages.
